# Giant Metastatic Gastrointestinal Stromal Tumor Occupying the Entire Abdominal Cavity Presenting as Transfusion-Dependent Anemia: A Comprehensive Case Report

**DOI:** 10.7759/cureus.115049

**Published:** 2026-08-23

**Authors:** Edgar Eduardo Sordia Marquez, Victor Hugo Garzón Ortega, Alec Anceno Olivares, Fernando Fernández Varela Gómez, Javier González Reyes

**Affiliations:** 1 Department of General Surgery, Christus Muguerza Hospital Alta Especialidad, Monterrey, MEX; 2 Department of Urology, University of Southern California, Mexico City, MEX; 3 Department of Plastic and Reconstructive Surgery, Instituto Nacional de Ciencias Médicas y Nutrición Salvador Zubirán, Mexico City, MEX

**Keywords:** abdominal tumor, case report, gastrointestinal stromal tumor, giant gist, gist, metastatic gist, multiorgan failure, sarcoma

## Abstract

Gastrointestinal stromal tumors (GISTs) are the most common mesenchymal neoplasms of the gastrointestinal tract and are usually diagnosed before reaching an advanced size. Giant GISTs exceeding 20 cm are uncommon and are frequently associated with adverse pathological features, including high mitotic activity, necrosis, and metastatic dissemination, which together drive an aggressive clinical course and a poor prognosis. We report the case of a 57-year-old man with type 2 diabetes mellitus who presented with progressive abdominal distension, fatigue, early satiety, transfusion-dependent anemia, and general deterioration. Computed tomography demonstrated a 29 × 20 cm intra-abdominal mass occupying nearly the entire abdominal cavity, without radiologic evidence of major vessel invasion. Given the severity of the anemia and the rapid clinical decline, the multidisciplinary team proceeded directly to exploratory laparotomy without preoperative tissue diagnosis; the rationale for this decision is discussed. Complete macroscopic tumor resection was achieved together with resection of a densely adherent segment of ileum and mechanical side-to-side intestinal anastomosis, but the procedure was complicated by an estimated 3,000 mL of blood loss, attributed to tumor neovascularity and adhesiolysis, requiring massive transfusion. Histopathology confirmed a 30 cm mixed-type GIST weighing more than 5 kg, with diffuse CD117 positivity, a mitotic index of 18/5 mm², focal necrosis, negative surgical margins (R0), and metastatic implants in the omentum and parietal peritoneum, consistent with high-risk metastatic disease without transmural bowel-wall invasion -- a pattern more in keeping with a mesenteric/extraintestinal GIST closely applied to the ileum than a primary transmural ileal tumor. Despite maximal postoperative intensive care, the patient developed refractory distributive shock, severe metabolic acidosis, and progressive multiorgan failure, and died on postoperative day 1. This case illustrates the extreme end of the GIST clinical spectrum and underscores the importance of early diagnosis, prompt referral, and structured multidisciplinary planning, including a deliberate, documented decision process around preoperative biopsy and neoadjuvant therapy, before disease reaches this magnitude.

## Introduction

Gastrointestinal stromal tumors (GISTs) are the most common mesenchymal tumors of the gastrointestinal tract and originate from the interstitial cells of Cajal or their precursors [1,2]. Most harbor activating mutations in KIT or PDGFRA, resulting in constitutive tyrosine kinase signaling that drives tumor growth [3]. Although GISTs typically arise within the muscularis propria of the stomach or small intestine, a minority arise primarily outside the bowel wall -- within the mesentery, omentum, or retroperitoneum -- and are classified as extragastrointestinal stromal tumors (EGIST); these lesions share the immunophenotype of conventional GIST but can present as very large masses before a bowel-wall origin is excluded [4,5].

Clinical presentation varies with tumor size and location. Small lesions are frequently asymptomatic and discovered incidentally, whereas larger tumors may present with abdominal pain, gastrointestinal bleeding, anemia, early satiety, weight loss, abdominal distension, or a palpable mass [5]. Tumor size, mitotic index, tumor rupture, and metastatic dissemination are the principal prognostic determinants; lesions larger than 10 cm with elevated mitotic activity carry a significantly increased risk of recurrence and disease-specific mortality [6]. Giant GISTs exceeding 20 cm remain rare and are generally reported as isolated cases or small series [7].

Current guidelines favor histologic confirmation -- by endoscopic ultrasound-guided fine-needle biopsy or, when the mass is not endoscopically accessible, image-guided percutaneous biopsy -- before committing a patient to either neoadjuvant imatinib or upfront surgery, because treatment selection depends on confirming a KIT/PDGFRA-driven tumor and excluding other diagnoses such as lymphoma or sarcoma. However, this pathway assumes a clinically stable patient who can tolerate the several weeks typically required to see a measurable response to neoadjuvant tyrosine kinase inhibition. In patients with acute, transfusion-dependent anemia and rapid functional decline, the safety of that delay is not established, and biopsy of a massive, heterogeneous, partially necrotic mesenteric mass carries its own risks of non-diagnostic sampling, bleeding, and, in rare instances, needle-tract tumor seeding. We present a giant, high-risk metastatic GIST measuring 30 cm and weighing more than 5 kg, associated with peritoneal implants and a fatal postoperative course despite complete macroscopic resection, and we use this case to examine that preoperative decision explicitly.

## Case presentation

A 57-year-old man with type 2 diabetes mellitus presented with progressive abdominal distension, fatigue, early satiety, and general deterioration over approximately six weeks. He denied abdominal pain, nausea, vomiting, overt gastrointestinal bleeding (hematemesis, melena, or hematochezia), bowel obstruction, or respiratory symptoms. The gradual, six-week pattern of decline and the absence of any reported acute bleeding episode were most consistent with chronic, low-grade occult blood loss from the tumor surface rather than a single acute hemorrhagic event, although endoscopic evaluation to exclude a luminal bleeding source was not performed (discussed further under the section Preoperative Diagnostic Strategy).

**Figure 1:**
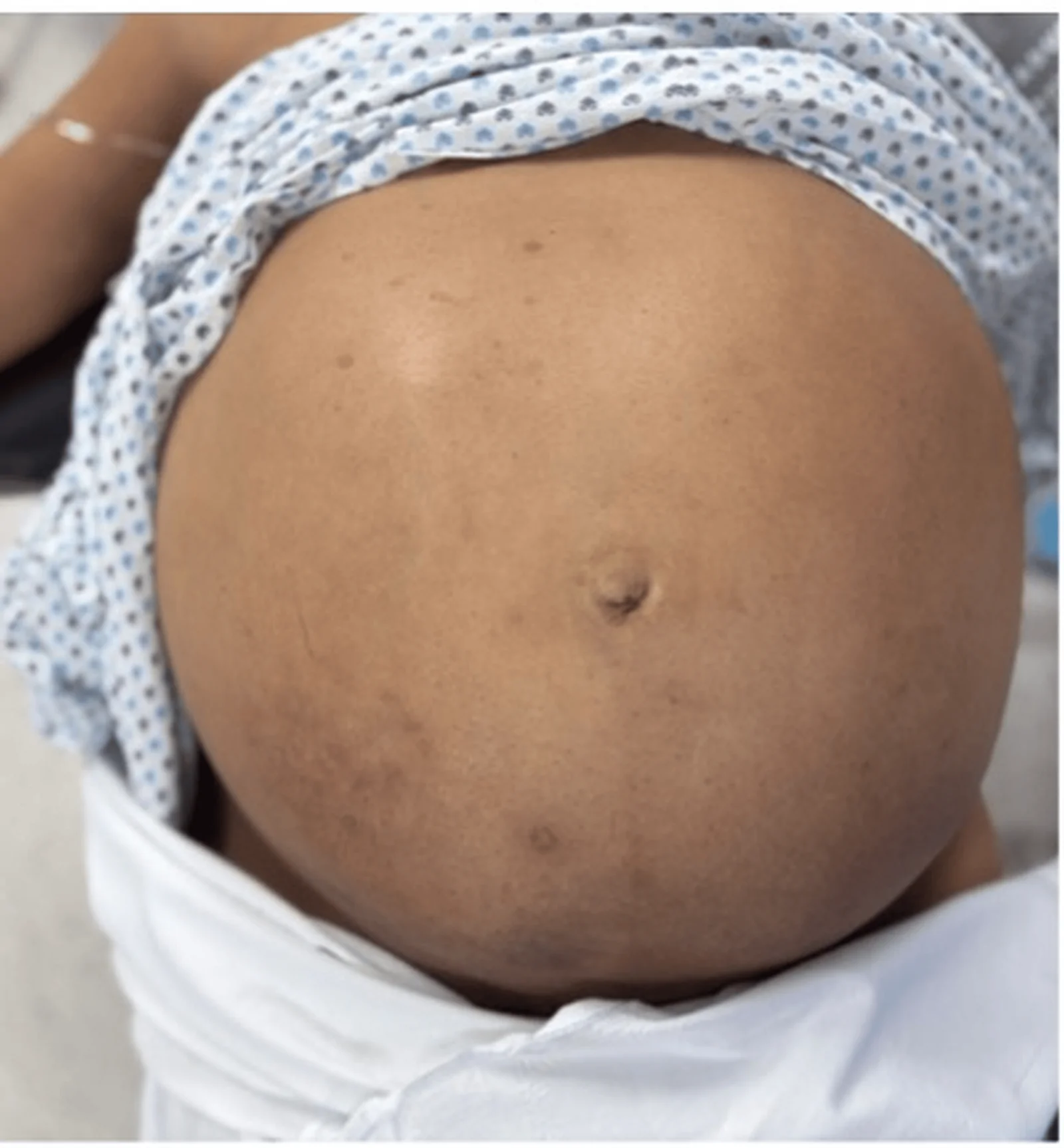
Clinical presentation Preoperative clinical photograph demonstrating marked abdominal distension secondary to a giant intra-abdominal gastrointestinal stromal tumor. The abdomen exhibited near-spherical enlargement with significant increase in abdominal girth, reflecting the extraordinary tumor burden occupying most of the abdominal cavity.

Physical examination revealed a markedly distended abdomen without signs of peritoneal irritation. A large abdominal mass occupying most of the abdominal cavity was clinically evident (Figure 1). The remainder of the examination was unremarkable.

Laboratory evaluation demonstrated severe symptomatic anemia. Hemoglobin levels progressively declined from 7.0 g/dL before admission to 6.9 g/dL on hospital day 1, requiring transfusion support. Two units of packed red blood cells were transfused preoperatively, with hemoglobin rising from 6.9 g/dL to approximately 8.8 g/dL.

**Figure 2:**
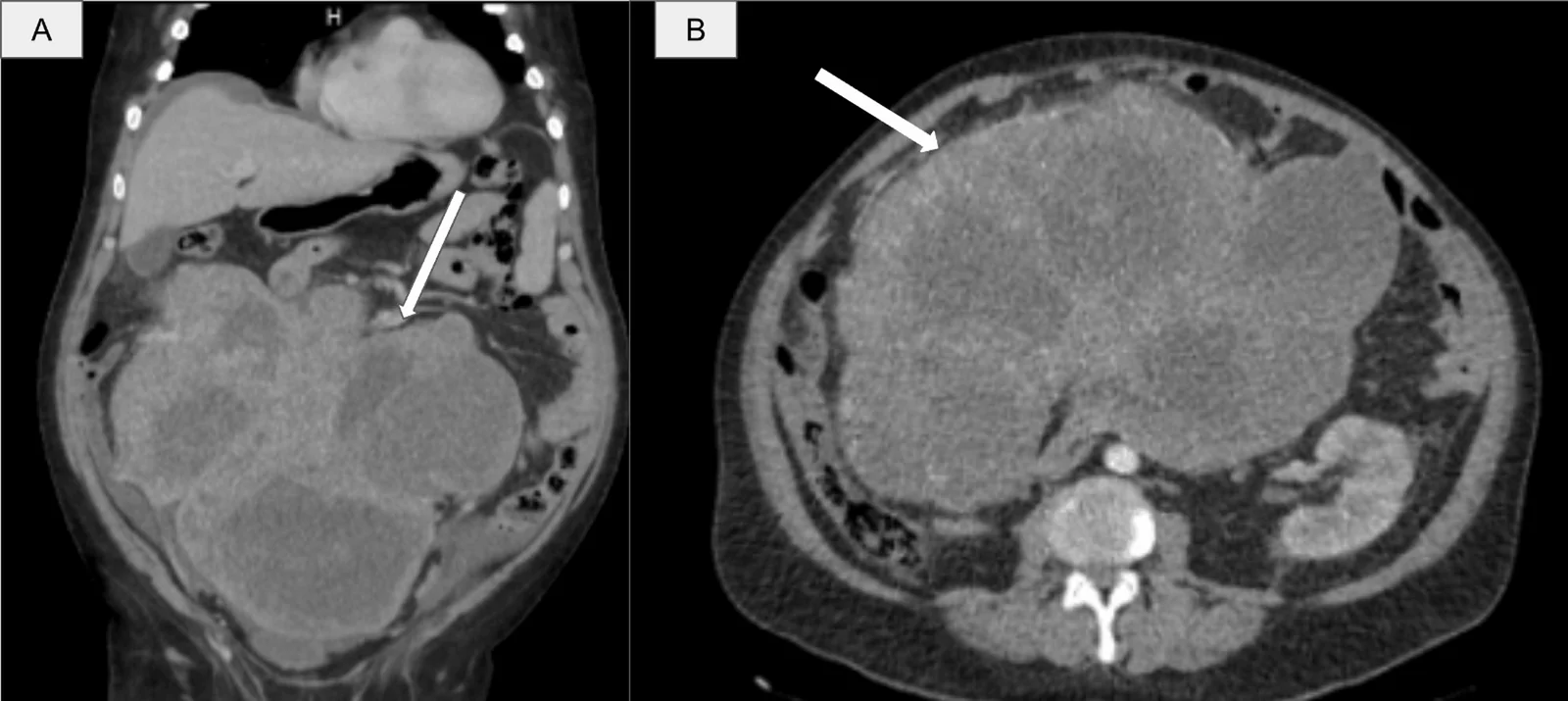
Contrast-enhanced computed tomography (A) Coronal reconstruction showing the cranio-caudal extension of the tumor and near-complete occupation of the abdominal cavity with displacement of surrounding bowel loops. (B) Axial view demonstrating the tumor measuring approximately 29 × 20 cm and producing significant mass effect on adjacent abdominal structures. Arrows indicate the tumor.

Contrast-enhanced computed tomography demonstrated a giant heterogeneous abdominal mass measuring approximately 29 × 20 cm, occupying a substantial portion of the abdominal cavity and producing significant mass effect on surrounding structures (Figure 2). The study showed no evidence of invasion or encasement of the aorta or other major retroperitoneal vessels. 

Given the tumor size, progressive symptoms, worsening functional status, and severe anemia, surgical management was indicated.

**Figure 3:**
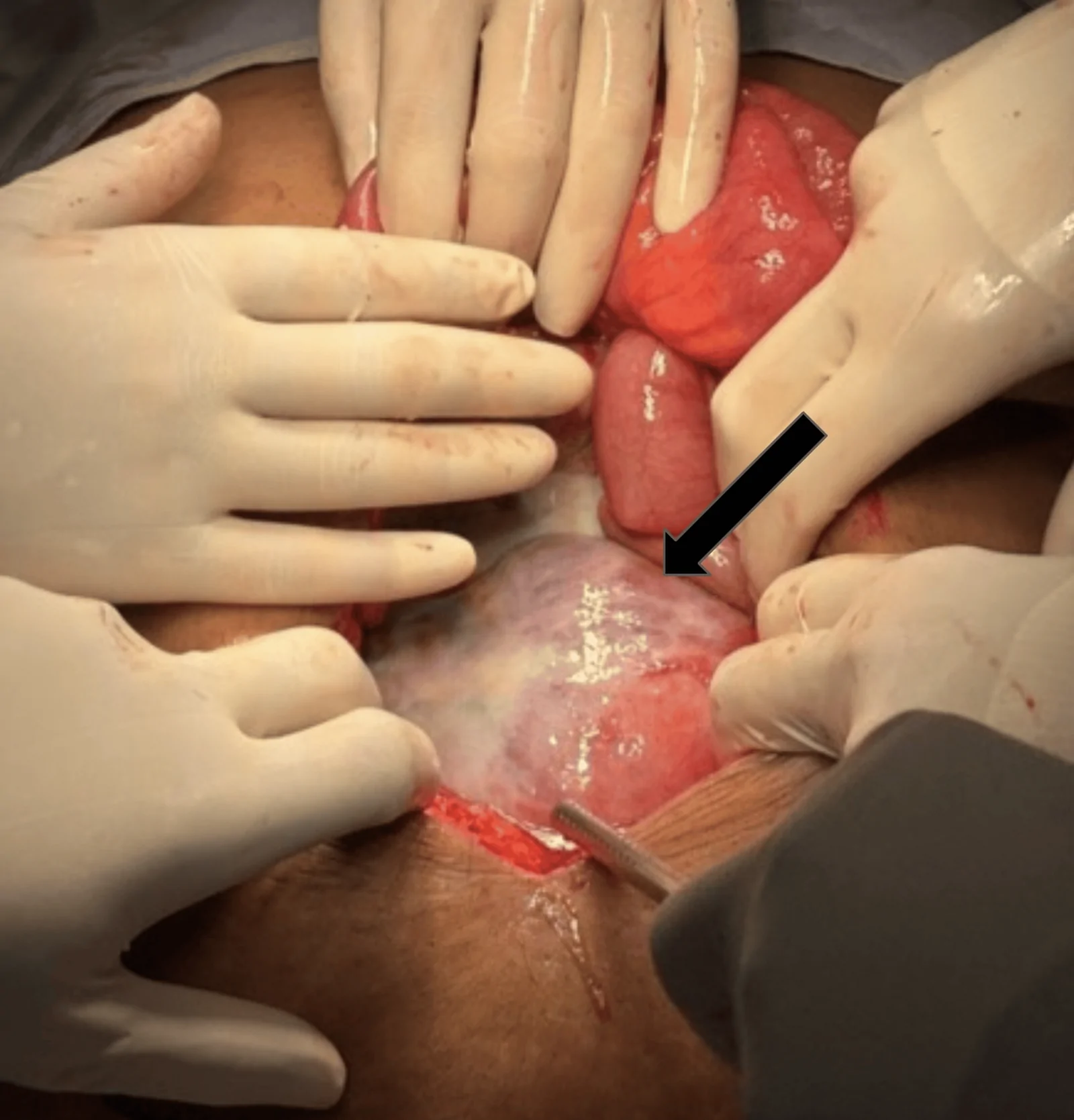
Intraoperative findings Intraoperative appearance of the giant abdominal tumor following laparotomy. The lesion occupied nearly the entire abdominal cavity and demonstrated extensive vascularity and adherence to adjacent intestinal segments. The arrow indicates the tumor surface.

On hospital day 1, exploratory laparotomy was performed through a midline incision, chosen to provide wide exposure for a mass occupying nearly the entire abdominal cavity and to allow rapid proximal vascular control if needed. A giant intra-abdominal neoplasm occupying nearly the entire abdominal cavity was identified (Figure 3). The tumor's macroscopic origin was the small bowel mesentery; it displaced, rather than clearly infiltrated, the surrounding intestinal loops, and no invasion of the aorta or other major retroperitoneal vessels was identified intraoperatively. Dense fibrovascular adherence between the tumor and an adjacent segment of ileum precluded safe separation and necessitated intestinal resection; this adherence was judged intraoperatively to represent dense adhesion rather than confirmed transmural invasion, an impression later corroborated by histopathology.

**Figure 4:**
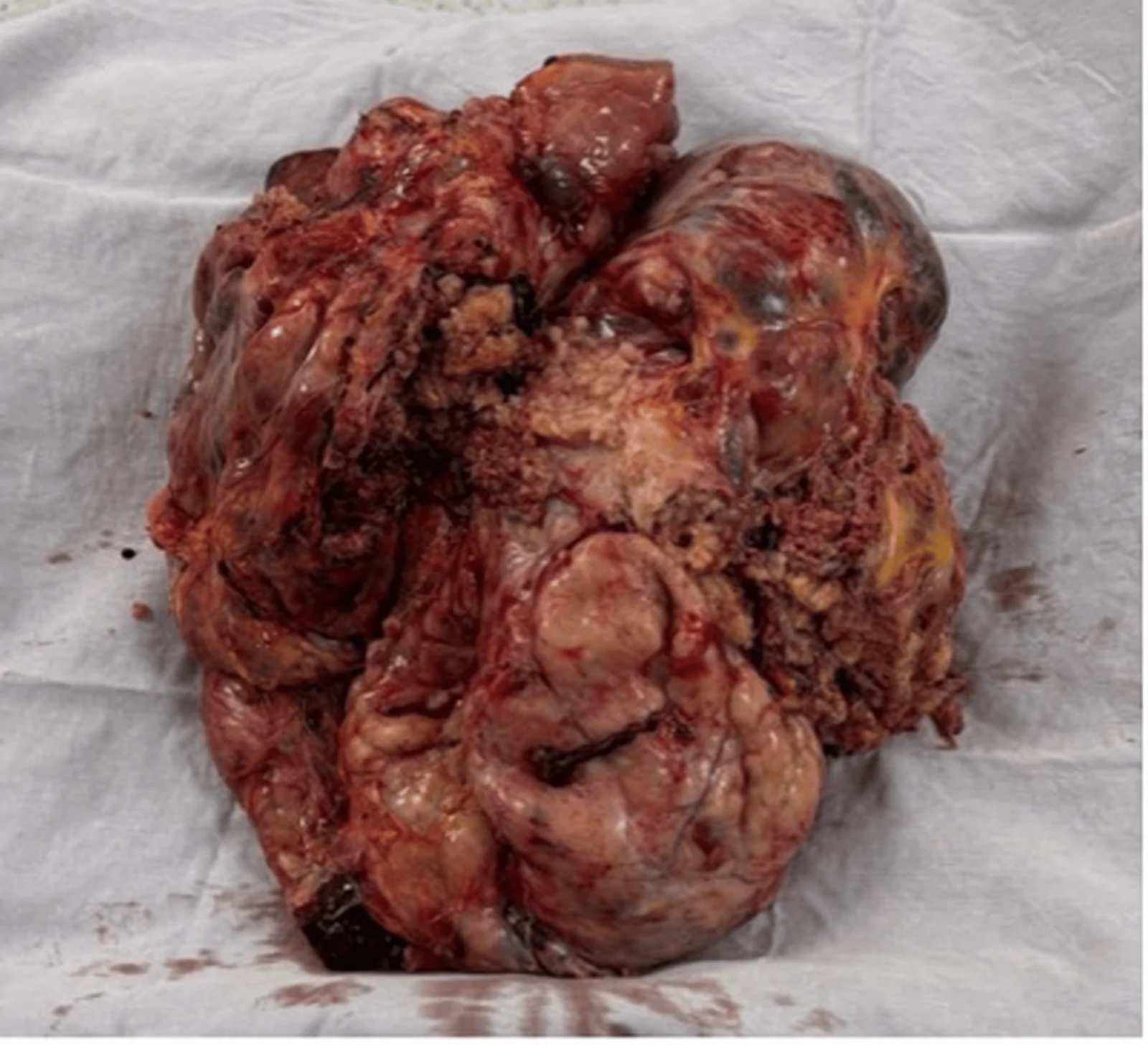
Surgical specimen Surgical specimen immediately after en bloc resection. The tumor measured 30.0 × 29.3 × 16.5 cm and weighed more than 5 kg, demonstrating a multinodular external surface with areas of necrosis.

Complete macroscopic excision was achieved through en bloc resection of the mass together with a 40 cm segment of ileum. Bowel continuity was restored with a mechanical side-to-side ileo-ileal anastomosis. The resected specimen weighed more than 5 kg (Figure 4). Total operative time was 4 hours. Estimated blood loss was approximately 3,000 mL, attributed to extensive tumor neovascularity and to adhesiolysis of the dense fibrovascular attachments between the tumor and the adjacent bowel and mesentery; no intraoperative tumor capsule rupture and no injury to a named major vessel were identified. Intraoperative transfusion included four units of packed red blood cells and one unit of fresh frozen plasma.

Following surgery, the patient was transferred to the intensive care unit under invasive mechanical ventilation and vasopressor support with norepinephrine, at a maximum dose of 1.0 mcg/kg/min. Despite aggressive resuscitation, his clinical condition deteriorated rapidly. Arterial blood gas analysis demonstrated profound metabolic acidosis, with a pH of 7.01, bicarbonate of 5.8 mmol/L, and lactate exceeding 15 mmol/L. Refractory shock, anuria, and progressive multiorgan dysfunction subsequently developed; postoperative serum creatinine peaked at 6.7 mg/dL. Abdominal drain output totaled 80 mL, and hemoglobin trended from 8.4 g/dL immediately postoperatively to 9.25 g/dL before death.

Broad-spectrum antimicrobial therapy, insulin infusion, vasopressor support, and advanced critical care measures were continued; however, no clinical recovery was achieved. The patient died on postoperative day 1 from refractory distributive shock following major abdominal tumor resection.

Histopathological and immunohistochemical findings

Gross examination revealed a giant tumor specimen weighing more than 5 kg and measuring 30.0 × 29.3 × 16.5 cm. The lesion was multinodular and partially encapsulated, with prominent surface vascularity. Sectioning demonstrated a predominantly solid neoplasm with a brown-red cut surface, extensive areas of soft friable tissue, and foci of yellow discoloration corresponding to necrosis, involving approximately 10% of the lesion.

Microscopic examination demonstrated a mesenchymal neoplasm with mixed spindle-cell and epithelioid components arranged in fascicular and storiform patterns. Tumor cells showed oval nuclei with mild-to-moderate atypia, eosinophilic cytoplasm, and increased mitotic activity, with a mitotic index of up to 18 mitoses per 5 mm² (20 high-power fields), supporting a high-grade lesion. Areas of vascular congestion and ischemic necrosis were also identified.

The tumor was intimately associated with the adjacent segment of ileum. Histologic examination of the intestinal specimen showed preserved mucosal architecture with only mild, chronic, nonspecific ileal inflammation and no transmural bowel-wall invasion. Together with the intraoperative impression of a mesenteric origin, this finding supports classification of the neoplasm as a mesenteric/extraintestinal GIST closely applied to, but not arising from within the wall of, the ileum, rather than a primary transmural ileal GIST.

**Figure 5:**
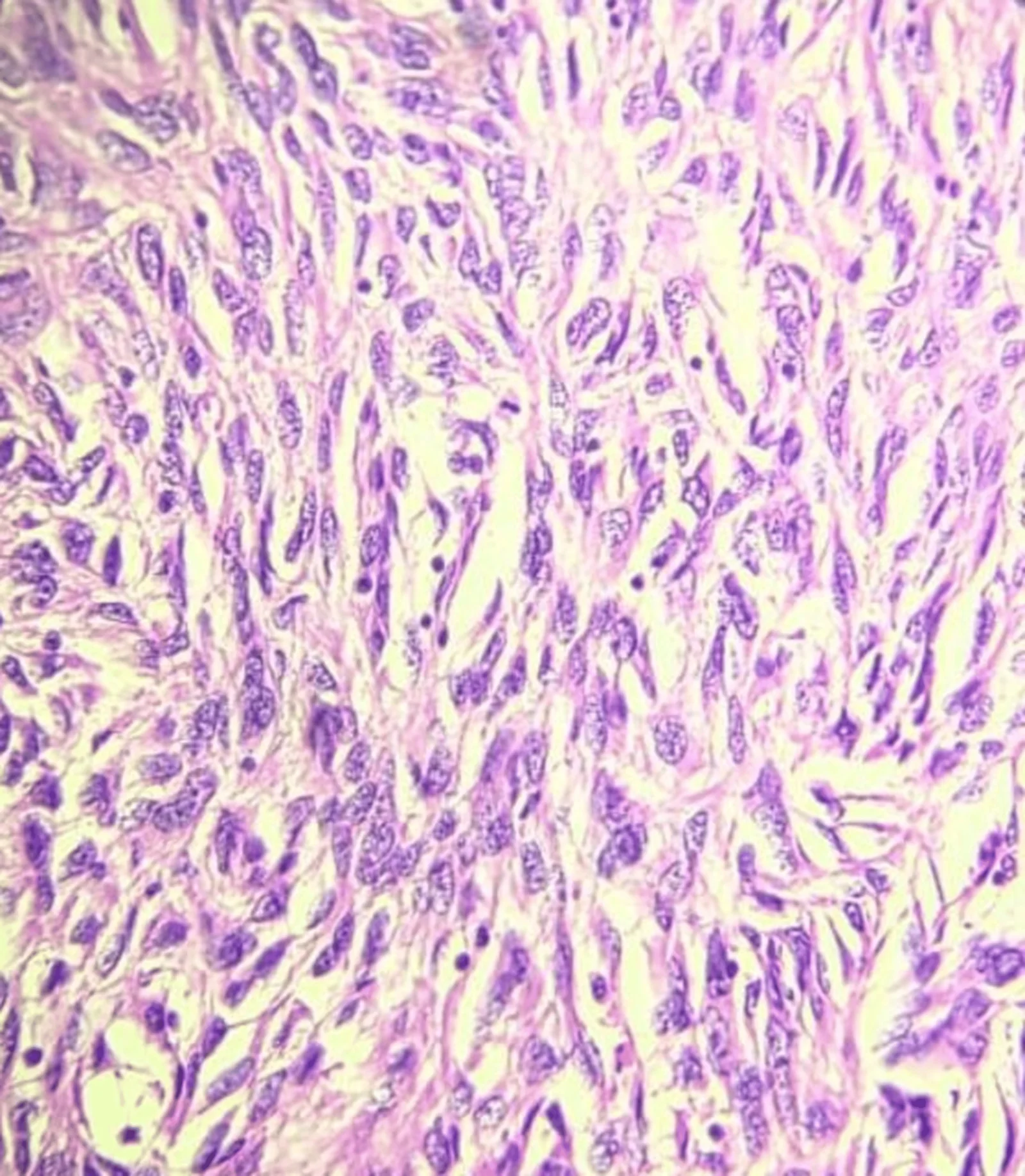
Histopathological findings Histopathological examination (H&E stain) demonstrated a spindle-cell neoplasm composed of elongated cells arranged in intersecting fascicles with eosinophilic cytoplasm and elongated nuclei, exhibiting the characteristic morphology of a spindle-cell gastrointestinal stromal tumor.

Immunohistochemistry demonstrated diffuse positivity for CD117 (KIT), while pancytokeratin, HMB45, S100, CD34, TLE1, and smooth muscle actin were negative. This morphologic and immunophenotypic profile is diagnostic of a GIST of mixed subtype (Figure 5).

Surgical margins were free of tumor, indicating complete macroscopic and microscopic resection (R0). Separate specimens from the omentum and parietal peritoneum demonstrated metastatic implants with identical histologic features, confirming intraperitoneal dissemination. According to the AJCC 8th edition staging system [8], the tumor was classified as pT4 pM1. Given its size, elevated mitotic activity, necrosis, and metastatic peritoneal implants, the lesion was categorized as a high-risk metastatic GIST.

## Discussion

Giant GISTs exceeding 20 cm remain exceptionally uncommon [1,2]. This tumor measured 30 cm in maximum diameter and weighed more than 5 kg, placing it among the largest reported in the contemporary literature [7]. Tumor size greater than 10 cm, high mitotic activity, necrosis, and metastatic dissemination are independently associated with aggressive behavior and unfavorable outcome [6,9]; the combination of 18 mitoses per 5 mm² and intraperitoneal implants in this patient established a diagnosis of advanced, high-risk metastatic disease. Diffuse CD117 positivity confirmed activation of the KIT pathway, present in roughly 95% of GISTs [3,10], and is the biological basis for tyrosine kinase inhibitor therapy in this disease [11].

Preoperative diagnostic strategy

International guidelines generally favor histologic confirmation before treatment, particularly when neoadjuvant imatinib is being considered for a large or borderline-resectable tumor, because response typically requires several weeks and because confirming KIT/PDGFRA status guides the choice and dose of therapy [11,12]. In this patient, that pathway was not pursued: the mass was predominantly extraluminal and mesenteric, which limits the yield and safety of endoscopic sampling; the patient's transfusion-dependent anemia and rapid functional decline argued against a delay of the weeks required to see a neoadjuvant response; and the patient's limited financial resources restricted access to additional preoperative diagnostic studies, a constraint common in resource-limited settings that is not always reflected in guideline-based algorithms [11,12]. We recognize this as a genuine trade-off rather than a straightforward decision. In retrospect, and in less acutely deteriorating patients with a similarly massive mesenteric mass and adequate access to diagnostic resources, image-guided core-needle biopsy -- accepting the small risks of non-diagnostic sampling or needle-tract seeding -- together with earlier multidisciplinary tumor-board discussion of neoadjuvant downsizing, represents the preferred pathway where the clinical trajectory and available resources allow it [11,12]. This case illustrates the opposite end of that spectrum, in which both the urgency of presentation and socioeconomic constraints removed that option.

Surgical approach and perioperative planning

A midline laparotomy was chosen to provide wide exposure of a mass occupying nearly the entire abdominal cavity and to allow rapid proximal vascular control if needed; no aortic or major vessel invasion was identified on preoperative imaging or intraoperatively. Giant intra-abdominal sarcomas and GISTs carry a substantial risk of major hemorrhage from tumor neovascularity and from adhesiolysis of dense fibrovascular attachments to adjacent viscera, independent of any true vascular invasion, as occurred in this case [13]. Reported strategies to mitigate this risk include preoperative cross-matching for massive transfusion, early availability of a vascular surgery team, judicious use of proximal vascular control maneuvers when major vessels are in proximity to the tumor, and staged or planned combined resections in anticipated multivisceral cases [13]. 

Although complete resection was achieved with negative margins, peritoneal implants indicated disseminated disease at presentation. Current guidelines recommend multidisciplinary management combining surgery and targeted therapy in selected patients with advanced GIST [11,12]. This patient's catastrophic postoperative deterioration precluded initiation of adjuvant or systemic treatment.

The fatal outcome was most likely multifactorial: massive tumor burden, extensive surgical manipulation, substantial blood loss, systemic inflammatory activation, and tissue hypoperfusion probably converged to produce the profound metabolic acidosis, hyperlactatemia, and multiorgan failure observed postoperatively. While perioperative mortality after GIST resection is uncommon in specialized centers, giant metastatic tumors carry a substantially higher operative risk [13].

The degree of preoperative anemia was itself a notable feature of this case. Hemoglobin values between 6.9 and 7.6 g/dL reflect the physiological burden imposed by chronic tumor-related blood loss and likely reduced the patient's reserve during the perioperative period [14].

Limitations

This report describes a single patient, and the preoperative and perioperative decisions described here were shaped by an acute, rapidly evolving clinical picture that limits generalization. The absence of preoperative tissue diagnosis, discussed above, means that a KIT/PDGFRA mutational profile -- which could have informed prognosis and, had the patient survived, adjuvant therapy selection -- was not available before surgery. 

## Conclusions

We report a giant, high-risk metastatic GIST measuring 30 cm and weighing more than 5 kg, with a mesenteric origin closely applied to the ileum, peritoneal dissemination, and a fatal postoperative course. Despite R0 resection, the patient developed refractory distributive shock, severe metabolic acidosis, and multiorgan failure, and died within the first postoperative day.

This case illustrates the extreme end of the biological and clinical spectrum of GIST, and highlights a genuine and often underdiscussed tension in giant abdominal masses: the standard preference for preoperative tissue diagnosis and, where appropriate, neoadjuvant therapy, versus the need for urgent surgical intervention in a patient with transfusion-dependent anemia and rapid functional decline. Early recognition of GIST before this degree of tumor burden develops, structured multidisciplinary planning that explicitly documents the biopsy/neoadjuvant decision, and proactive preparation for massive intraoperative hemorrhage are the principal lessons we draw from this case.
